# Roles of type 1 regulatory T (Tr1) cells in allergen-specific immunotherapy

**DOI:** 10.3389/falgy.2022.981126

**Published:** 2022-08-03

**Authors:** Masaya Matsuda, Tetsuya Terada, Kazuyuki Kitatani, Ryo Kawata, Takeshi Nabe

**Affiliations:** ^1^ Laboratory of Immunopharmacology, Faculty of Pharmaceutical Sciences, Setsunan University, Hirakata, Japan; ^2^ Department of Otolaryngology, Head & Neck Surgery, Osaka Medical and Pharmaceutical University, Takatsuki, Japan

**Keywords:** allergy, allergen, interleukin-10, immunotherapy, Tr1 cells

## Abstract

Allergen-specific immunotherapy (AIT) is the only causative treatment for allergic diseases by modification of the immune response to allergens. A key feature of AIT is to induce immunotolerance to allergens by generating antigen-specific regulatory T (Treg) cells in allergic patients. Type 1 regulatory T (Tr1) cells and forkhead box protein 3 (Foxp3)-expressing Treg cells are well known among Treg cell subsets. Foxp3 was identified as a master transcription factor of Treg cells, and its expression is necessary for their suppressive activity. In contrast to Foxp3^+^ Treg cells, the master transcription factor of Tr1 cells has not been elucidated. Nevertheless, Tr1 cells are generally considered as a distinct subset of Treg cells induced in the periphery during antigen exposure in tolerogenic conditions and can produce large amounts of anti-inflammatory cytokines such as interleukin-10 and transforming growth factor-β, followed by down-regulation of the function of effector immune cells independently of Foxp3 expression. Since the discovery of Tr1 cells more than 20 years ago, research on Tr1 cells has expanded our understanding of the mechanism of AIT. Although the direct precursors and true identity of these cells continues to be disputed, we and others have demonstrated that Tr1 cells are induced in the periphery by AIT, and the induced cells are re-activated by antigens, followed by suppression of allergic symptoms. In this review, we discuss the immune mechanisms for the induction of Tr1 cells by AIT and the immune-suppressive roles of Tr1 cells in AIT.

## Introduction

Breaking immunotolerance to innocuous antigens causes the development of allergic diseases, such as asthma, rhinitis, and conjunctivitis. The development of allergic diseases is mainly dominated by type 2 immunity. Allergen-specific Th2 cells and type 2 innate lymphoid cells (ILC2) have essential roles in the development by producing type 2 cytokines, such as interleukin (IL)-4, IL-5 and IL-13 (1). These cytokines induce allergen-specific IgE antibody production from B cells and eosinophilic infiltration and proliferation, leading to exacerbation of allergic symptoms (1). Epithelial cells are also regulated by type 2 cytokines. IL-4 orchestrates epithelial cells toward type 2 phenotype (E2 phenotype), characterized by upregulation of chemokine ligand 26 (CCL26) and IL-24 expressions, which induce the production of mucus and anti-microbial peptides (2), leading to the development of airway remodeling (3). Conversely, interferon-γ (IFN-γ) impedes the acquisition of the E2 phenotype on epithelial cells (2). Although understanding of the mechanisms underlying allergic diseases has been advanced (4), pharmacotherapies including molecular targeted drugs have been developed for the treatment (5), the prevalence of allergic diseases has gradually increased worldwide (6, 7). Therefore, modification of the natural history of allergic diseases is crucial for a radical cure of allergic diseases.

Allergen-specific immunotherapy (AIT) is the only causative treatment for allergic diseases by induction of immune tolerance to allergens (8). Since Noon (9) first demonstrated that subcutaneous injection of a grass pollen extract was effective in modulating sensitivity to grass pollen, the mechanisms and development of further safe treatment routes have been elucidated (10). Currently, AIT has been mainly conducted in two forms: subcutaneous immunotherapy (SCIT) and sublingual immunotherapy (SLIT) (11). The clinical effectiveness and safety of SCIT and SLIT for allergic diseases, especially allergic rhinitis (12, 13) and asthma (14, 15), have been established. Moreover, AIT can prevent not only the development of allergic diseases (16) but also sensitization to new allergens (17). However, AIT remains underused mainly because (1) long-term treatment is required to acquire sustainable remission of allergic symptoms (18, 19), (2) some patients are non-responders (20, 21) and (3) rare anaphylactic reactions (22, 23). Therefore, a deeper understanding of the mechanisms associated with AIT is essential in order to develop more effective treatments.

The mechanisms of AIT have been dissected in different compartments: (1) B cell-associated changes (24): induction of regulatory B cells, an increase in allergen-specific IgG4 antibodies, and a decrease in allergen-specific IgE antibodies, (2) ILC2-related changes (25, 26): immune deviation from ILC2 toward ILC1, decreases in IL-5 and IL-13 productions, and inversely an increase in IL-10 production, and (3) T-cell associated changes (27–30): immune deviation from Th2-cell toward Th1-cell response, suppression of T follicular helper cells, trans-differentiation from Th17 cells into T regulatory 17 (Tr17) cells, and increases in regulatory T (Treg) cells. Concurrent with Treg cells by AIT, studies (31–33) have demonstrated that AIT induced T-cell exhaustion in humans and mice. Interleukin (IL)-10 and transforming growth factor (TGF)-β, which are produced by AIT-induced Treg cells, have been demonstrated to enforce T-cell exhaustion (34, 35). On the other hand, another AIT study (36) reported that the exhaustion markers, programmed cell death-1 (PD-1) and cytotoxic T-lymphocyte-associated protein 4 (CTLA-4), on Th2 cells were decreased in the up-dosing of AIT, but persisted for long-term during the maintenance phase of the treatment. Therefore, the induction of Treg cells by AIT could be crucial for maintaining immunotolerance to allergens.

Although various subsets of Treg cells have been reported, CD4^+^ Treg cells are well-characterized. In addition, the existence of CD8^+^ Treg cells (37), CD4^−^ CD8^−^ Treg cells (38), and γδ Treg cells (39) has also been reported. As shown in Table 1, CD4^+^ Treg cells can be broadly classified into two groups based on where the cells occurred: thymus-derived Treg (tTreg) cells and peripherally induced Treg (pTreg) cells (40–47). tTreg cells constitutively express the transcription factor forkhead box protein 3 (Foxp3), which controls the immunosuppressive functions of Treg cells (48). After their maturation, they move to tissues to prevent harmful immune responses against self-antigens. On the other hand, pTreg cells develop from naïve CD4^+^ T cells when the cells are persistently exposed to exogenous antigens, followed by the induction of tolerance toward exogenous antigens such as allergens. Moreover, pTreg cells are divided into two subsets based on whether Foxp3 is expressed in the cells: Foxp3^+^ pTreg cells and Foxp3^−^ type 1 regulatory T (Tr1) cells.

**Table 1:** CD4^+^ regulatory T (Treg) subsets.

Subsets		Candidate markers	References	
			Human	Mouse
tTreg cells	Foxp3^+^ tTreg cells	Helios^high^ Nrp-1^high^	–	(40–42)
		CD45RA^+^ CD25^+^ Foxp3^+^ (Resting tTreg)	(43, 44)	–
		CD45RA^−^ CD25^high^ Foxp3^high^ (Activated tTreg)	(43, 44)	–
pTreg cells	Foxp3^+^ pTreg cells	Helios^low^ Nrp-1^low^	(45)	(42, 46)
		CD45RA^−^ CD25^+^ Foxp3^+^	(43, 44)	–
	Foxp3^−^ Tr1 cells	CD49b^+^ CD226^+^ LAG-3^+^ CD25^low^ CTLA4^low^	(47)	(47)

CTLA4, cytotoxic T-lymphocyte-associated protein 4; Foxp3, forkhead box protein 3; LAG-3, lymphocyte activation gene-3; Nrp-1, neuropilin-1; pTreg, peripherally induced Treg; tTreg, thymus-derived Treg; Tr1, type 1 regulatory T.

More than 30 years ago, an original study on human leukocyte antigen (HLA) fully mismatched fetal liver hematopoietic stem cells successfully transplanted into a patient with severe combined immunodeficiency (SCID) led to the groundbreaking discovery of Tr1 cells (49). In this patient, immunological tolerance with mixed chimerism of donor T cells and recipient antigen-presenting cells was developed without immunosuppressive agents. However, T cells derived from this patient significantly proliferated upon exposure to host antigens *in vitro*. This result suggested the presence of active suppression mechanisms in peripheral tissues. The human interleukin (IL)-10 gene was subsequently isolated from CD4^+^ T cells derived from this successfully transplanted patient (50). Moreover, host-specific IL-10-producing CD4^+^ T cells were identified in an another successfully transplanted patient (51). Groux et al. (52) demonstrated that IL-10-producing CD4^+^ T cells can be generated by chronic antigen stimulation in the presence of IL-10 in mice and humans. These antigen-specific IL-10-producing CD4^+^ T cells effectively suppressed the development of colitis induced in SCID mice and were named Tr1 cells (52). A subsequent study (53) demonstrated that Tr1 cells exerted immunosuppressive functions independent of Foxp3.

Many studies have been reported that latency-associated peptide (LAP)^+^ CD25^−^ CD4^+^ T cells (54, 55), natural killer group 2, member D (NKG2D)^+^ CD25^−^ CD4^+^ T cells (56, 57), CD127^low^ CD25^+^ CD4^+^ T cells (58), CD49b^+^ CD25^−^ CD4^+^ T cells (59–62), lymphocyte activation gene 3 (LAG3)^+^ CD25^−^ CD4^+^ T cells (63), CD44^high^ CD62L^low^ IL-7 receptor (IL-7R)^−^ LAG3^+^ CD49b^+^ LAP^+^ CD4^+^ T cells (64), and C-C chemokine receptor type 5 (CCR5)^+^ programmed cell death 1 (PD-1)^+^ CD25^−^ CD4^+^ T cells (65, 66) were Tr1 cells in mice or humans (Table 2). Recently, Gagliani et al. (47) demonstrated that CD49b- and LAG3-expressing CD4^+^ T cells can be identified as the true phenotype of Tr1 cells in mice and humans. On the other hand, Huang et al. (67) reported that co-expression of CD49b and LAG3 were not restricted to Foxp3^−^ Tr1 cells but also observed in Foxp3^+^ Treg cells. In this way, the specific markers of Tr1 cells continue to be disputed.

**Table 2:** Phenotypes of Tr1 cells in humans and mice.

Subsets	References	
	Human	Mouse
CD49b^+^ CD226^+^ LAG3^+^ CD25^low^ CTLA4^low^ CD4^+^ T cells	(47)	(47)
CD44^high^ CD62L^low^ IL-7R^−^ LAG3^+^ CD49b^+^ LAP^+^ CD4^+^ T cells	–	(64)
CD49b^+^ CD25^−^ CD4^+^ T cells	–	(59–62)
LAG3^+^ CD25^−^ CD4^+^ T cells	(63)	–
CCR5^+^ PD-1^+^ CD25^−^ CD4^+^ T cells	(65, 66)	–
LAP^+^ CD25^−^ CD4^+^ T cells	–	(54, 55)
NKG2D^+^ CD25^+^ CD4^+^ T cells	(56)	(57)
CD127^low^ CD25^+^ CD4^+^ T cells	(58)	–

CCR5, C-C chemokine receptor type 5; IL-7R, IL-7 receptor; LAG3, lymphocyte activation gene 3; LAP, latency-associated peptide; NKG2D, natural killer group 2, member D; PD-1, programmed cell death 1.

Although there is a lack of specific markers of Tr1 cells, the existence of various allergen-specific Tr1 cells whose characteristic features are Foxp3-negative IL-10-producing CD4^+^ T cells, have also been reported in allergic models of mice (68–70) and allergic patients (71–78). Several clinical studies (79–82) demonstrated that the number of Tr1 cells increased in AIT-treated allergic patients, and the number such cells correlated with the clinical score. These studies suggested that Tr1 cells have crucial roles in immune tolerance to allergens. In this review, we focused on Tr1 cells, and discussed the immune mechanisms for the induction of Tr1 cells and the immune-suppressive roles of Tr1 cells in AIT.

## Clinical relevance of Tr1 cells in allergy and AIT

Breakdown of immunological tolerance to allergens results in the development or exacerbation of allergic diseases. One of the features in the breakdown of immunological tolerance is the reduction or dysfunction of Tr1 cells. Studies (74, 76) have reported that the numbers of Tr1 cells in allergic patients was significantly lower than those in healthy subjects. Our group also revealed that the numbers of Tr1 cells in peripheral blood of Japanese cedar pollinosis patients was markedly lower than those of healthy subjects (81). Han et al. (76) demonstrated that the frequency of *Dermatophagoides pteronyssinus* major allergen 1 (Der p 1)-specific Tr1 cells in peripheral blood was decreased in house dust mite-induced allergic rhinitis patients, and it correlated with the clinical symptom scores. Moreover, the dysfunction of Tr1 cells was observed in allergic patients. CD46 is a complement regulatory protein that is upregulated in activated leukocytes to protect these cells from autologous complement-induced lysis at inflammatory sites (83). Cross-linking of CD46 during T-cell stimulation leads to the strong induction of Tr1 cells (84). Xu et al. (71) first demonstrated that the number of CD46-induced Tr1 cells was decreased in the peripheral blood of asthmatic patients compared with that of healthy subjects. The failure of a generation of Tr1 cells was observed in asthma but also multiple sclerosis (85). Ni Choileain et al. (86) demonstrated this failure was caused by the incorrect recruitment of CD46 to the immunological synapse, which reduced the conversion of effector CD4^+^ T cells to Tr1 cells. More recently, it has been shown that peanut-specific Tr1 cells can be induced in healthy subjects and patients with peanut allergies, but those in patients with peanut allergies are functionally defective (73). Therefore, the lack of the generation and function of allergen-specific Tr1 cells led to the development and deterioration of allergic diseases in humans.

In vivo induction of allergen-specific Tr1 cells has been explored to acquire immune tolerance to allergens. Repetitive administration of bee venom through bee stings in non-allergic beekeepers transformed from bee venom-specific Th2 cells into Tr1 cells (72). These Tr1 cells suppressed the proliferation of bee venom-specific T cells *in vitro*. This result suggested that AITs may be effective for allergic diseases because of the conversion of allergen-specific effector Th2 cells into tolerance-inducing Tr1 cells by repeated allergen administrations. Indeed, many studies [reviewed in (10, 87)] have demonstrated that AIT can be effective for various allergic diseases, and the frequency of allergen-specific Tr1 cells was correlated with the decrease in clinical scores (79–82). Taken together, these studies suggested that Tr1 cells play a critical role in the induction of immunotolerance to allergens in humans.

Several studies (88–90) demonstrated that not only Tr1 cells but also Foxp3^+^ Treg cells were increased in AIT-treated patients with allergies. Although which subset of Tr1 cells or Foxp3^+^ Treg cells is important for the improvement of allergic symptoms in AIT has not been fully proven, it may be dependent on the route of antigen administration. Our group previously demonstrated that the number of Tr1 cells but not Foxp3^+^ Treg cells was increased in peripheral blood and inflamed tissues of SCIT-treated allergic patients and mice (70, 81). Moreover, the induced Tr1 cells produced a large amount of IL-10 in response to antigen stimulation, followed by suppression of the development of asthma in the OVA-induced airway inflammation model of mice (91, 92). Lou et al. (79) also reported that the increase in Tr1 cells by SCIT was correlated with improvement in nasal symptoms, but the number of Foxp3^+^ Treg cells was not. These data imply that the increase in Tr1 cells but not Foxp3^+^ Treg cells could be crucial for the induction of immunotolerance to allergens by SCIT. On the other hand, our group (82) reported that both numbers of Tr1 cells and Foxp3^+^ Treg cells were markedly increased in the peripheral blood of Japanese cedar pollinosis patients who had received SLIT. Moreover, the number of Foxp3^+^ Treg cells positively correlated with improvement in nasal symptoms, whereas those of Tr1 cells did not (82). Therefore, Foxp3^+^ Treg cells rather than Tr1 cells may have contributed to the clinical effects of SLIT. This is also supported by a study of Xian et al. (93). Therefore, which subset of Tr1 cells or Foxp3^+^ Treg cells is more relevant to clinical tolerance may depend on the antigen administration route.

## Induction mechanisms of Tr1 cells in AIT

**Figure 1:**
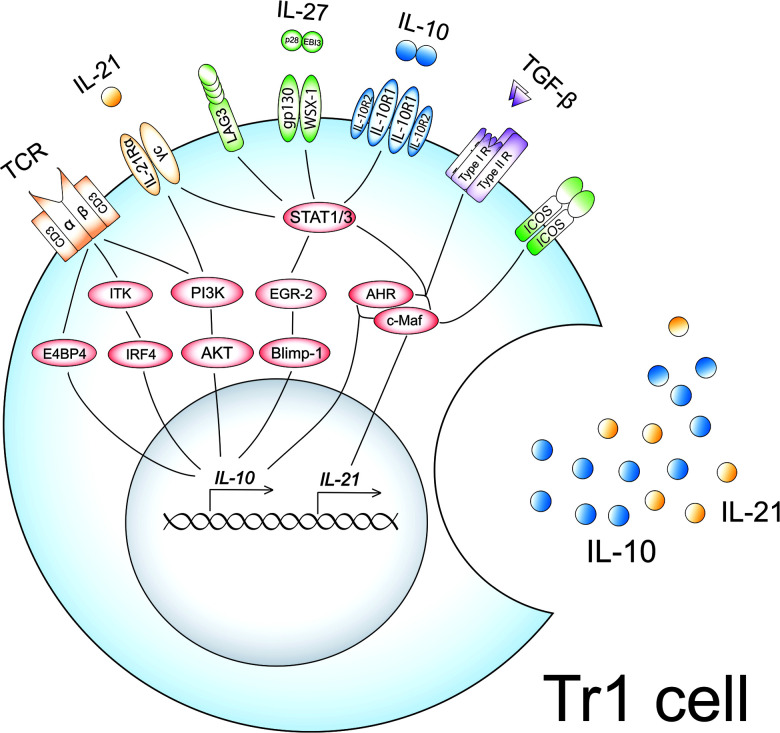
Induction mechanisms of Tr1 cells in AIT. High-dose allergen exposure in AIT promotes dendritic cells to produce IL-27, IL-10, and TGF-β. Naïve CD4^+^ T cells can differentiate into Tr1 cells upon TCR engagement in the presence of these cytokines. High-dose antigen exposure leads to the upregulation and activation of IL-10-associated molecules such as ITK, E4BP4, IRF4, PI3K, and AKT, followed by the production of IL-10. PI3K also enforces IL-21 receptor expression. IL-27 upregulates and activates IL-10-associated molecules, EGR-2, Blimp-1, AHR, and c-Maf, followed by the production of IL-10 and IL-21 in cooperation with TGF-β *via* activation of STAT1 and STAT3. IL-27 also upregulates the expression of ICOS and LAG3. The upregulated IL-10, IL-21, and ICOS by IL-27 amplify the productions of IL-10 and IL-21 in an autocrine manner, leading to the acquisition of a Tr1 phenotype.

High-dose allergen exposure in AIT promotes dendritic cells to produce IL-27, IL-10, and transforming growth factor-beta (TGF-β) (94, 95). Naïve CD4^+^ T cells can differentiate into Tr1 cells upon T cell receptor (TCR) engagement in the presence of these cytokines. The induction mechanisms of Tr1 cells in AIT are summarized in Figure 1.

Several reports (91, 96) demonstrated that antigen-specific stimulation was crucial for the generation of Tr1 cells. We also revealed that the number of Tr1 cells was increased significantly when spleen cells isolated from ovalbumin (OVA)-sensitized mice were cultured with a high concentration of OVA (10^−3^ g/ml) for 7 days (91). On the other hand, the induction of Tr1 cells was not observed in a culture of spleen cells derived from non-sensitized mice (91). Motomura et al. (96) also reported that chronic antigen-stimulated Th1 cells into IL-10-producing Tr1 cells *via* up-regulation of a transcription factor of IL-10, E4-binding protein 4 (E4BP4) in mice. One study demonstrated that the expression of E4BP4 on CD4^+^ T-cell populations was markedly up-regulated after AIT in mice (32). Our group also reported that the expression of E4BP4 mRNA on peripheral blood mononuclear cells in SCIT-treated Japanese cedar pollinosis patients was higher than those in non-SCIT-treated patients (81). These data suggested that antigen-specific stimulation may be essential for the induction of Tr1 cells *via* upregulation of E4BP4.

IL-2 inducible T cell kinase (ITK) is a non-receptor tyrosine kinase mainly observed in T cells and has a vital role in the TCR signaling (97). ITK signaling is critical for T-cell subset differentiation and the regulation of cytokine gene expression. Huang et al. (98) demonstrated that the induction of Tr1 cells was not observed in the absence of ITK in mice and humans. ITK deficiency impaired interferon regulatory factor 4 (IRF4) expression, leading to the underdevelopment of Tr1 cells in humans and mice. Overexpression of IRF4 rescued the development of these cells in ITK-deficient cells derived from both humans and mice. These findings suggest that ITK signaling components are essential for developing Tr1 cells.

The phosphatidylinositol-3 kinase (PI3K)-protein kinase B (AKT) signaling, which is a downstream of TCR activation, is also involved in the induction and maintenance of Tr1 cells. Once activated, PI3K-AKT-signaling by TCR engagement in the presence of IL-27 resulted in both upregulation of IL-10 and IL-21 receptors on naive CD4^+^ T cells in mice (99). These upregulated molecules are crucial for the induction and maintenance of Tr1 phenotypes, as described below in this paper. The authors also reported that FoxO1 phosphorylation was dampened in the condition of suppression of the PI3K and AKT phosphorylation, leading to impairment of Tr1 cell differentiation by IL-27 (99). Although the roles of FoxO1 in Tr1 differentiation have not been fully elucidated, FoxO1 was described as a critical molecule for the induction of IL-10, TGF-β, and CTLA-4 on CD4^+^ T cells in mice (100, 101). These reports suggested that PI3K-AKT-FoxO1 signaling could be crucial for the acquisition of Tr1 cell phenotypes.

IL-27 is a pleiotropic cytokine that is a heterodimer composed of Epstein-Barr virus-induced gene 3 (Ebi3) and IL-27 p28 (102). IL-27 is mainly produced by antigen-presenting cells upon Toll-like receptor stimulation (102, 103) or chronic antigen stimulation (104). IL-27 binds to the IL-27 receptor, a heterodimer composed of the orphan cytokine receptor WSX-1 and a signal transducing chain, glycoprotein 130 (gp130) (105). IL-27 induces the activation of STAT1 and STAT3, leading to the up-regulation of transcription factors such as c-Maf and aryl hydrocarbon receptor (AHR). c-Maf (106) and AHR (107) directly transactivated IL-10 gene expression through binding to a xenobiotic response element motif and a c-Maf recognition element motif in the IL-10 promoter, respectively. The interaction of AHR with c-Maf synergistically induces not only IL-10 but also IL-21 production, which is the hallmark of Tr1 cells (108). IL-27 also induces early growth response gene 2 (Egr-2) expression in naïve CD4^+^ T cells *via* activation of STAT3 (109). Egr-2 has been reported to be the transcription factor that binds to the B lymphocyte-induced maturation protein-1 (Blimp-1) promoter, leading to the upregulation of Blimp-1 (109). Blimp-1 binds to intron 1 of the *IL10* locus, leading to the production of IL-10 from mouse CD4^+^ CD25^+^ Treg cells (110). Egr-2 also enforces the expression of lymphocyte activation gene 3 (LAG-3) (109). Moreover, the expression of inducible T-cell co-stimulator (ICOS) was increased in IL-27-stimulated naïve CD4^+^ T cells (111). ICOS is a coreceptor molecule, a member of the CD28 family, that is induced in activated T cells (112). The expression of ICOS-ligand (ICOS-L) is found in dendritic cells, macrophages, and CD4^+^ T cells, and the expression is further amplified upon activation of these cells (113, 114). ICOS induced the upregulation of c-Maf, followed by the production of IL-10 and IL-21 (115). Therefore, IL-27 is important for initiating the differentiation of Tr1 cells *via* up-regulation of STAT1 and STAT3.

The produced IL-10 and IL-21 from induced Tr1 cells are involved in the maintenance of phenotypes of Tr1 cells in an autocrine manner. As mentioned above, IL-10 is an anti-inflammatory cytokine that forms a homodimer and exerts its function through binding to its receptor. The IL-10 receptor consists of two subunits of IL-10R1 and two subunits of IL-10 receptor 2 (IL-10R2) (116). IL-10 only binds to IL-10R1 but not IL-10R2 (117). The IL-10 binds to its receptor, followed by the activation of STAT1 and STAT3. IL-21 is a potent immunomodulatory four-alpha-helical bundle type I cytokine that has pleiotropic roles in the regulation of T-cells, B-cells, natural killer cells, and myeloid cells (118). The functional receptor of IL-21 is composed of the IL-21Rα chain and the common cytokine receptor γc chain (119). IL-21 binds to its receptor, leading to the activation of STAT3. STAT3 activated by IL-10 and IL-21 also enforces the expression of c-Maf, leading to the maintenance of Tr1 phenotypes. TGF-β is also an immunomodulatory cytokine that exacerbates the induction of Tr1 cells *via* upregulation of c-Maf and AHR in the presence of IL-27 (106, 108, 120).

Our group (91, 92) also demonstrated that the stimulation of allergens, IL-21, IL-27, and TGF-β of CD4^+^ T cells have crucial roles in the induction of allergen-specific Tr1 cells in mice. Spleen cells isolated from OVA-sensitized mice were cultured in the presence of OVA, IL-21, IL-27, and TGF-β for 7 days. After 7 days of culture, a significant increase in Foxp3-negative IL-10-producing CD4^+^ T cells was observed in the culture. Moreover, most of the induced Foxp3-negative IL-10-producing CD4^+^ T cells were double positive for CD49b and LAG3, which are surface markers of Tr1 cells. Therefore, the antigen-presenting environment in the presence of IL-21, IL-27, and TGF-β may be a suitable condition for the induction of allergen-specific Tr1 cells.

In recent years, Zissler et al. (121) reported that AIT induced upregulations of secretoglobin1A1 (SCGB1A1) and IL-7 in the airway of patients with allergic rhinitis. These factors are produced by airway epithelial cells (121, 122) and could be involved in the induction of Tr1 cells. SCGB1A1 is mainly produced by airway club cells, which are a kind of epithelial cells (123). Mandal et al. (124) demonstrated that SCGB1A1 down-regulated cyclooxygenase-2 gene expression in airway epithelial cells of asthmatic mice, followed by decreases in the production of prostaglandins (PGs). Hooper et al. (125) reported that PGE_2_ inhibited Tr1 cell differentiation by suppressing IL-27 production from dendritic cells in mice. These data suggested that SCGB1A1 is associated with the induction of Tr1 cells *via* suppressing the production of PGE_2_ at inflammatory milieus. IL-7 is a cytokine that is required for the development and maintenance of most major subsets of T cells (126). IL-7 also supports Tr1 cell proliferation in cooperation with IL-2 and IL-15 (127). Therefore, AIT could modulate the phenotypes of epithelial cells, leading to the induction of Tr1 cells in the inflamed tissues.

RNA interference by microRNA (miRNA) has also been associated with the mechanisms of AIT. Jakwerth et al. (128) demonstrated that the expression of miR-3595, whose target is *PTGER*, prostaglandin EP_3_ receptor, was markedly up-regulated in the sputum of AIT-treated patients with allergic rhinitis. Moreover, it was reported that the level of its ligand PGE_2_ in the sputum was decreased by AIT-treatment (128). As mentioned above, PGE_2_ signaling suppresses the induction of Tr1 cells. These data suggested that AIT could suppress the PGE_2_-EP3 axis *via* a decrease in PGE_2_ production and downregulation of EP3, leading to the induction of Tr1 cells.

Extracellular matrix components such as hyaluronan (HA) are also involved in the induction and maintenance of Tr1 cells. The biological functions of HA are dependent on its size (129). Although the low molecular weight of HA promotes antigen presentation and allergic responses, intact high molecular weight HA (HMW-HA) has anti-inflammatory properties and contributes to healing tissues (129). Bollyky et al. (130) demonstrated that HMW-HA promoted the induction of Tr1 cells from effector memory T-cell precursors *via* binding its receptor, CD44, in humans and mice. The produced IL-10 from Tr1 cells also enforces HMW-HA production from fibroblast *via* activating the STAT3-dependent signaling pathway in mice (131, 132). Therefore, the crosstalk of Tr1 cells and extracellular matrix such as HMW-HA could be essential for wound healing in inflamed tissues. The combination SLIT with HMW-HA markedly suppressed the development of airway hyperresponsiveness in comparison with the only SLIT-treated asthmatic mice (133). Gebe et al. (134) also reported that modified HMW-HA, which was thiolated and tethered to OVA *via* thiol linkages, enforced allergen-specific immune tolerance *via* IL-10 upregulation in the lungs of OVA-induced asthmatic model of mice. These data imply that HMW-HA is an attractive adjuvant for AIT.

## Immunosuppressive roles of Tr1 cells

**Figure 2:**
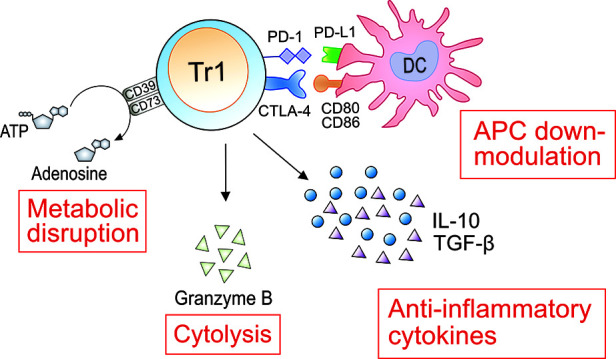
Immunosuppressive roles of Tr1 cells in AIT. As immunosuppressive mechanisms by activated Tr1 cells, (1) suppression of effector cells by anti-inflammatory cytokines, (2) down-modulation of antigen-presenting cells (APCs) by immune checkpoint molecules, (3) cytolysis of effector cells by granzyme B, and (4) metabolic disruption by CD39 and CD73 have been clarified. IL-10 can suppress the production of IL-5 and IL-13 from Th2 and ILC2. IL-10 and TGF-β downregulate the expression of major histocompatibility complex class II molecules and co-stimulatory molecules such as CD80 and CD86, and the production of pro-inflammatory cytokines by antigen-presenting cells, followed by suppression of activation of effector T cells. Tr1 cells suppress the functions of Th2 by down-modulating APCs through expression of CTLA-4 and PD-1. Granzyme B produced from Tr1 cells induces apoptosis of the interacted APCs. Adenosine produced by CD39 and CD73-expressed on Tr1 cells restrained the functions of Th2 cells and ILC2. The induced Tr1 cells by AIT are involved in the suppression of allergic symptoms using these four mechanisms.

Tr1 cells need to be activated by specific antigen recognition *via* their TCR. Once activated by a specific antigen, Tr1 cells can also suppress the activities of other antigen-specific T cells in their proximity. Therefore, Tr1 cells mediate both antigen-specific and antigen-non-specific immune reactions. However, the antigen-non-specific immunosuppression is restricted to the tissue where these cells are activated (135). As immunosuppressive mechanisms by activated Tr1 cells, (1) suppression of effector cells by anti-inflammatory cytokines, (2) down-modulation of antigen-presenting cells by immune checkpoint molecules, (3) cytolysis of effector cells by granzyme B, and (4) metabolic disruption by CD39 and CD73 have been demonstrated (Figure 2). These suppressive mechanisms have been reported both for murine and human Tr1 cells (Table 3).

**Table 3:** Immunosuppressive roles of Tr1 cells in humans and mice.

Mechanisms of suppression	References	
	Human	Mouse
Anti-inflammatory cytokines (IL-10 and TGF-β)	(52, 139)	(69, 170, 171)
Cytolysis (Granzyme B)	(151, 152, 172–174)	–
APC down-modulation (PD-1 and CTLA-4)	(74, 154)	–
Metabolic disruption (CD39 and CD73)	(158–160)	(161)

APC, antigen-presenting cell; CTLA-4, cytotoxic T-lymphocyte-associated protein 4; IL-10, interleukin-10; PD-1, programmed cell death 1; TGF-b, transforming growth factor-β.

Upon being activated by a specific antigen, Tr1 cells produce large amounts of anti-inflammatory cytokines, especially IL-10 and TGF-β in both mice and humans (47). We previously demonstrated that the adoptive transfer of Tr1 cells significantly suppressed the development of airway hyperresponsiveness and increased levels of eosinophils and neutrophils in the lung *via* the production of a large amount of IL-10 in an asthmatic model in mice (91, 92). Other studies (136, 137) demonstrated that IL-10 significantly inhibited the upregulation of vascular adhesion molecule-1 (VCAM-1) and intercellular adhesion molecule-1 (ICAM-1) on endothelial cells and leukocyte adhesion to endothelium. Our group also demonstrated that the intratracheal administration of IL-10 suppressed the infiltration of eosinophils and neutrophils into the lung *via* down-regulation of the expression of VCAM-1 and ICAM-1 on pulmonary vascular endothelial cells in severely asthmatic mice (138). IL-10 also induces the downregulation of the expression of major histocompatibility complex class II molecules and co-stimulatory molecules such as CD80 and CD86, and the production of pro-inflammatory cytokines by antigen-presenting cells, followed by suppression of activation of effector T cells (139). IL-10 can also directly suppress the production of type 2 cytokines such as IL-4, IL-5, and IL-13 from Th2 cells (140) and group 2 innate lymphoid cells (ILC2) (141). TGF-β also down-regulates the expression of CD80 and CD86 on dendritic cells, followed by inhibition of the interaction with effector T cells (142). In recent years, Branchett et al. (143) demonstrated that TGF-β suppressed the production of CCL8, a chemokine of Th2 and ILC2, from alveolar macrophages in a house dust mite-induced allergic airway inflammation model in mice. In addition to suppressing the functions of effector cells, TGF-β can elicit Foxp3 expression in naïve CD4^+^ T cells (144). However, TGF-β inversely induces the differentiation of naïve CD4^+^ T cells into Th9 cells (145) and Th17 cells (146) in the presence of pro-inflammatory cytokines, such as IL-4 and IL-6. Even upon allergen exposure, TGF-β1 was described as a crucial accelerator of induction of Th2, Th9 and Th17 cells in a recent publication (147). On the other hand, we previously demonstrated that TGF-β1 amplified the differentiation of Tr1 cells upon allergen exposure in cooperation with IL-27 and IL-21 in mice (91). Moreover, Th17 cell induction was not observed in this culture condition (91). As mentioned above, large amounts of IL-21, IL-27 and TGF-β1 could be produced in the milieu of Tr1 induction by AIT. Therefore, TGF-β1 may be associated with Tr1 induction rather than inflammatory Th cell differentiation in AIT.

On the other hand, many studies (51, 148–150) reported that the addition of neutralizing antibodies against IL-10 and TGF-β did not completely abrogate the suppressive functions of human Tr1 cells. These data suggested that a cell contact-dependent mechanism was associated with inhibiting immune responses by Tr1 cells. Magnani et al. (151) demonstrated that human Tr1 cells were activated by binding CD54, CD58, and CD155 on antigen-presenting cells *via* their own expression of lymphocyte function-associated antigen 1, CD2, and CD226, respectively, followed by the production of granzyme B. The produced granzyme B from Tr1 cells induces apoptosis of the interacting antigen-presenting cells. This cytolysis of antigen-presenting cells leads to suppression of both antigen-specific CD4^+^ T cells and CD8^+^ T cells, and non-specific T cells (152). In mice, although the expression of granzyme B on Tr1 cells was reported (153), the contribution to the immunosuppressive ability of Tr1 cells has not been elucidated.

Tr1 cells can also inhibit the activation of effector cells *via* binding immune checkpoint molecules such as PD-1 and CTLA-4. Chen et al. (154) demonstrated that blocking CTLA-4 or PD-1/ programmed cell death ligand 1 almost completely abolished human Tr1 cell-mediated inhibition of effector T cell proliferation. Akdis et al. (74) also reported that human Tr1 cells suppressed the production of IL-13 from Der p 1 or Bet v 1-specific Th2 by down-modulation of antigen-presenting cells through expressions of CTLA-4 and PD-1 on Tr1 cells. In mice, the expression of CTLA-4 (155, 156) and PD-1 (66, 157) on Tr1 cells has also been reported in mice, but their contribution to the immunosuppressive potential of Tr1 cells is unclear.

The expression of the ectonucleotidase CD39 and the ecto-5′-nucleotidase CD73 were observed on the surface of human Tr1 cells (158–160). In mice, the expression of CD39 but not CD73 was observed in mouse Tr1 cells (161). The extracellular adenosine triphosphates produced in inflammatory environments are sequentially hydrolyzed to 5′-adenosine monophosphate (AMP) and then to adenosine by CD39 and CD73 (162, 163). Adenosine binds to A1, A_2a,_ A_2b,_ and A_3_ receptors (R), which are expressed on the surface of various immune cells and non-immune cells (164). In immune cells, adenosine mainly binds to A_2a_R and A_2b_R, followed by suppressing the functions *via* upregulation of intercellular cAMP (165, 166). Csoka et al. (167) demonstrated that the interaction of adenosine with A_2a_R suppressed the development of Th2 cells from naïve CD4^+^ T cells in mice. Moreover, Csoka's group (168) reported that A_2b_R had suppressive roles in the production of IL-5 and IL-13 from ILC2 in mice. Xiao et al. (169) also demonstrated that adenosine restrained ILC2-driven allergic airway inflammation *via* binding to A_2a_R in mice.

## Conclusion

Since the discovery of Tr1 cells, our understanding of the mechanisms of AIT has expanded through the constant efforts of many groups. As for the induction mechanisms of Tr1 cells in AIT, it has been clarified that three key events are essential for the induction and maintenance of these cells: (1) antigen presentation, (2) the production of IL-10, IL-27, and TGF-β from high-dose allergen-stimulated dendritic cells, and (3) the production of IL-10 and IL-21 from the induced Tr1 cells in an autocrine manner. When the induced Tr1 cells are reactivated by the specific antigen, the cells suppress effector cells by using anti-inflammatory cytokines, immune checkpoint molecules, granzyme B, and ectoenzymes such as CD39 and CD73. However, the master regulator of these cells is still unclear. The discovery of the master regulator of Tr1 cells may lead to the development of more-effective AIT. Therefore, understanding the biology of Tr1 cells should continue to be expanded.

## Author contributions

MM, TT, KK, RK and TN wrote and revised the manuscript. All authors contributed to the article and approved the submitted version.
